# The Potential Therapeutic Effect of RNA Interference and Natural Products on COVID-19: A Review of the Coronaviruses Infection

**DOI:** 10.3389/fphar.2021.616993

**Published:** 2021-02-23

**Authors:** Mohammad Reza Kalhori, Fatemeh Saadatpour, Ehsan Arefian, Masoud Soleimani, Mohammad Hosien Farzaei, Ina Yosifova Aneva, Javier Echeverría

**Affiliations:** ^1^ Medical Biology Research Center, Health Technology Institute, Kermanshah University of Medical Sciences, Kermanshah, Iran; ^2^ Molecular Virology Lab, Department of Microbiology, School of Biology, College of Science, University of Tehran, Tehran, Iran; ^3^ Department of Hematology, Faculty of Medical Sciences, Tarbiat Modares University, Tehran, Iran; ^4^ Medical Technology Research Center, Health Technology Institute, Kermanshah University of Medical Sciences, Kermanshah, Iran; ^5^ Institute of Biodiversity and Ecosystem Research, Bulgarian Academy of Sciences, Sofia, Bulgaria; ^6^ Departamento de Ciencias del Ambiente, Facultad de Química y Biología, Universidad de Santiago de Chile, Santiago, Chile

**Keywords:** coronavirus, miRNA, siRNA, natural products, phytochemicals, SARS-CoV-2

## Abstract

The SARS-CoV-2 virus was reported for the first time in Wuhan, Hubei Province, China, and causes respiratory infection. This pandemic pneumonia killed about 1,437,835 people out of 61,308,161cases up to November 27, 2020. The disease’s main clinical complications include fever, recurrent coughing, shortness of breath, acute respiratory syndrome, and failure of vital organs that could lead to death. It has been shown that natural compounds with antioxidant, anticancer, and antiviral activities and RNA interference agents could play an essential role in preventing or treating coronavirus infection by inhibiting the expression of crucial virus genes. This study aims to introduce a summary of coronavirus’s genetic and morphological structure and determine the role of miRNAs, siRNAs, chemical drugs, and natural compounds in stimulating the immune system or inhibiting the virus’s structural and non-structural genes that are essential for replication and infection of SARS-CoV-2.

## Introduction

Over the past 50 years, a wide range of human and animal diseases have been caused by coronaviruses (CoVs). Since the emergence of Severe Acute Respiratory Syndrome (SARS-CoV) Coronavirus in 2003, a considerable number of new Human coronaviruses (H-CoVs) have been identified (Ding et al., 2003). The appearance of Middle East Respiratory Syndrome (MERS-CoV) coronavirus in 2012 and the continuous occurrence of human cases further intensified attention to the importance of studying these viruses from different aspects (Centers for Disease Control and Prevention, 2012). Tolerance of new mutations and recombination has led to the evolution of this group of viruses, giving them the ability to transmit between species. In most cases, CoV infections are self-limiting, and after completing a reasonable period, the body recovers from the illness (Enjuanes et al., 2000). However, some strains of this family cause severe infections and have been the cause of widely spread epidemics during the last two decades (Mahase, 2020).

The SARS-CoV-2 virus was reported for the first time in Wuhan, Hubei Province, China, and causes respiratory infection. According to WHO statistics, on November 27, 2020, Coronavirus disease 2019 (COVID-19) killed about 1,437,835 people out of 61,308,161cases, and the most significant number of deaths and infected people were in the Americas (Chen et al., 2020b). In recent years, many treatment strategies such as prescribing antibiotics, the application of antiviral drugs (including Human Immunodeficiency Virus 1 (HIV-1) protease inhibitors, oseltamivir, and ribavirin), different corticosteroids, interferons, and natural human immunoglobulin have been used for treating patients suffering from H-COVs. Recently, most treatment strategies for CoVs such as MERS-CoV, SARS-CoV, and Severe Acute Respiratory Syndrome coronavirus-2 (SARS-CoV-2) have been based on inhibiting viral agents involved in the replication, infection, or induction of host immune system agents to combat viral infection (Li and De Clercq, 2020; Prajapat et al., 2020). Numerous studies are being conducted today to prove the role of various biomolecules, including RNA interference (RNAi), fusion inhibitors, neutralizing antibodies, plant, or microbial metabolites, as antiviral compounds (Prajapat et al., 2020).

This review article tries to present some information about the biology, replication, infusion, and pathogenesis of coronavirus. Also, it has decided to explain the existing treatment strategies based on chemical drugs, natural compounds, small interfering RNA (siRNAs), and microRNA (miRNAs) that can be used to fight against coronavirus infection, especially SARS-CoV-2. For this purpose, keywords including natural product, flavonoid, polyphenols, phytochemicals, microRNA, siRNA, Coronavirus, COVID-19 up to August 2020 were searched and evaluated using Scopus, PubMed, WOS, and Google Scholar databases.

## Coronavirus

### Coronavirus Classification and Taxonomy

Genome organization, genome homology, reproduction strategies in a host, and the virion’s structural traits are criteria for CoVs classification by the International Committee for Taxonomy of Viruses (ICTV) (Casals et al., 1980). In terms of phylogenetics, CoVs belong to Coronaviridae, as the class of Nidovirals. Coronaviridae family includes two sub-families: Orthocoronavirinae and Torovirinae. The sub-family of Orthocoronavirinae consists of four genera: Alpha, Beta, Gamma, and Delta coronaviruses that are responsible for infection on a vast range of hosts, from mammalians to birds (King et al., 2012). Human infection with CoVs was first reported in 1965 (Hamre and Procknow, 1966). H-CoV 229E and CoV-NL63 are human pathogens of the genus Alpha-CoV, causing common cold (Mcintosh et al., 1970; Monto, 1974). Severe Acute Respiratory Syndrome Coronavirus 1 (SARS-CoV-1), MERS-CoV, and SARS-CoV-2 are phylogenetically classified in the Beta-CoVs genus and have caused a high percentage of mortality in the human population during the last two decades (Zhou et al., 2020). The most common CoVs genes used for phylogenetic studies are nsp12 (RNA dependent RNA polymerase), nsp5 (chymotrypsin-like protease), nsp13 (helicase), nucleocapsid (N), and spike protein (S) (ICTV, 2009).

### Genomic Structure

CoVs are positive single-strand RNA with 32–46% G + C content. They have the biggest genome size among all known RNA viruses (about 26.4–31.7 kb) (Lai and Cavanagh, 1997; Enjuanes et al., 2000). Depending on the strain, the genome in CoVs contains a different number of open reading frames (ORF). Nevertheless, ORF1a, ORFb1, envelope (E), protein S, protein N, and membrane protein (M) are present in all Human-CoV strains (Cavanagh, 1995; Enjuanes et al., 2000). The SARS-CoV-2 genome size varies from 29.8 to 29.9 kb and encodes 9,860 amino acids. The ORF1ab in SARS-CoV-2 is over 21 KB in length and covers two-thirds of the entire genome (Cascella et al., 2020). According to recent reports, the ORF portion of the SARS-CoV-2 genome has a lower CG dinucleotide percentage than other coronaviruses, so ORF RNA translation is highly efficient (Wang Y. et al., 2020).

### Coronavirus Replication Process

**FIGURE 1:**
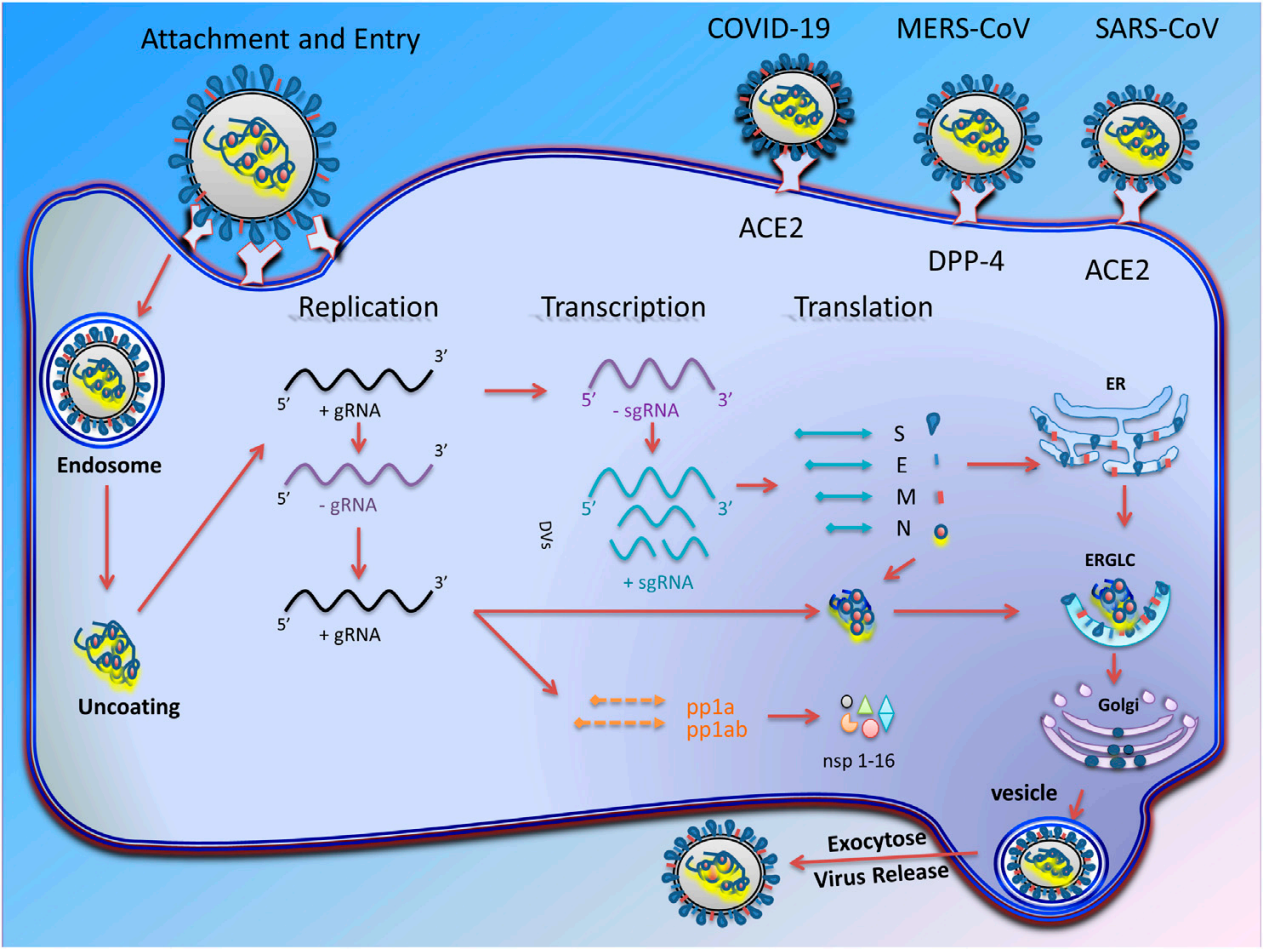
Transcription and replication of coronavirus. Schematic picture showing the coronavirus infection starts with attachment of virus S proteins to receptors on the host cells. After successful fusion, ribonucleocapsid enters the cell cytoplasm and loses its coat to mRNA is released. RNA-dependent RNA synthesis in CoVs includes two different genome replication processes to achieve multiple copies of genomic RNAs (gRNAs) and transcription of sub-genomic RNAs (sg mRNAs) coding structural and accessory proteins. The gRNA eventually producing 16 non-structural proteins (nsp-16) that are involved in virus replication. After assembly and budding, the full virus particles are transferred to the cytoplasmic membrane and finally released through the exocytosis process.

The replication cycle of CoVs occurs in several stages (Figure 1). In the initial stage, the virus binding proteins are attached to appropriate receptors on the host cell’s surface. Then membrane fusion occurs, which finally leads to virus genome insertion in the host cell. In the second step, the RNA polymerase-dependent RNA gene is activated to translate the virus genome into structural proteins (Ziebuhr, 2005). During these steps, in addition to viral factors, some host cell factors can also enhance or inhibit this process.

### Attachment; Entry and Cellular Factors Involvement

The first stage of virus entry starts with the attachment of S proteins to host ligands. Most HCoVs use endocytosis pathways for their entrance, while others can use direct membrane fusion (Gallagher and Buchmeier, 2001). The ectodomain S protein consists of a receptor-binding subunit (S1) and a membrane subunit (S2). After binding to the host cell surface receptors, the S1 subunit leads to a structural change in S2. The S2 proteolytic domain then fuses the virus membrane into the host membrane, leading to the viral genome’s entry into the host cell. A partial mutation in the S protein that changes the amino acid sequence can significantly affect the virus’s pathogenesis and tissue or cell susceptibility to infection (Li, 2016).

HCoV-NL63, SARS-CoV, and SARS-CoV-2, use Angiotensin-Converting Enzyme 2 (ACE2) to contaminate cells (Moore et al., 2004; Zhang et al., 2020a). Both types of alveolar cells (I and II types) and the endothelial cells express ACE2. It has been shown that MERS-CoV uses Dipeptidyl peptidase 4 (DPP4), a physiological ligand for adenosine deaminase, which is extensively expressed by the endothelial cells in various tissues (Raj et al., 2013). However, many host factors can limit the entry of the virus. According to cell culture mechanisms, a family of Interferon-Inducible Transmembrane proteins (IFITM) could limit S protein-dependent access in HCoV-229E and HCoV-NL63, which would also lead to intense reduction of the infection in SARS-CoV and MERS-CoV (Huang et al., 2011; Wrensch et al., 2014). It seems that IFITM inhibits the membrane fusion by preventing the virus fusion to the cell membrane, endosomal membranes, or through membrane fluidity. Moreover, it was shown that human coronavirus HCoV-OC43 utilizes IFITM2 and IFITM3 as receptors to facilitate cell entry (Zhao et al., 2014).

### Transcription and Replication

In CoVs, RNA-dependent RNA polymerase amplifies the virus genome in two ways and eventually produces several copies of genomic RNAs (gRNAs) and subgenomic RNAs (sg mRNAs) (Ziebuhr, 2005). Generally, CoVs replication is initiated with the translation of ORF1a to produce polyprotein1a (pp1a) with 4,382 amino acids and polyprotein1ab (pp1ab) with 7,073 amino acids. Then, the ORF1b is translated through the ribosomal frameshifting mechanism (Lai, 1990; Ziebuhr, 2005). Eventually, each of these polyproteins is cleaved to produce 16 non-structural proteins (nsp-16), which are involved in the virus replication cycle (Mcintosh and Peiris, 2009). Some of the non-structural proteins (Table 1) that have critical roles in the virus’s life cycle include helicase (nsp13), RNA-dependent RNA polymerase (RdRP) (nsp12 polymerase), 3-chymotripsin-like protease (3CL^pro^) (nsp5 protease), and papain-like protease (PL^pro^) (nsp3 protease) (Lai, 1990; Sawicki et al., 2005).

**TABLE 1:** Biological activities of coronavirus non-structural proteins.

Proteins	Biological functions in virus particle	Effect on the host cellular response	Refs.
Nsp1	A virulence factor.	Interact with the 40S subunit of ribosome to prevent host mRNA translation.	Woo et al. (2010)
	The genus-specific marker.		
	Highly divergent among CoVs		
Nsp2	Not reported.	Interaction with two host proteins, PHB1 and PHB2 and disruption of intracellular host cell surviving signaling pathway.	Cornillez-Ty et al. (2009)
Nsp3	Cysteine-type endopeptidase activity at the N-terminus of the replicase polyprotein.	Plays a role in host membrane rearrangement. Downregulate mRNA levels of pro-inflammatory cytokines including CCL5, and CXCL10.	Lei et al. (2018)
	Nucleic acid binding by interaction with N protein, RNA-directed 5′-3′ RNA-directed 5′-3′ RNA polymerase activity.	Responsible for suppressing IFN induction. Antagonize IFN production	
Nsp4	Marker for the coronavirus-induced DMVs.	Not reported.	Angelini et al. (2013)
	Interacts with nsp3 and nsp6 to formation of the replication complexes.		
Nsp5	Cleaves the C-terminus of replicase polyprotein at 11 sites	Bind an ADRP, may cleave host ATP6V1G1 and modifying host vacuoles intracellular pH	Lin et al. (2005)
Nsp6	Necessary for viral replication.	Plays a role in the initial induction of autophagosomes from host reticulum endoplasmic.	Angelini et al. (2014)
	Mediates the DMV formation by rearrangement of host membrane.	Limits the expansion of these phagosomes that are no longer able to deliver viral components to lysosomes.	
Nsp7	A primase in the form of heterohexadecamer dsRNA-encircling as ring structure.	Not reported.	Te Velthuis et al. (2012)
Nsp8	A processivity factor for the RdRP.		
Nsp9	May participate in viral replication by acting as a ssRNA-binding protein.	Not reported.	Miknis et al. (2009)
Nsp10	Interact with nsp1, nsp7, nsp14, and nsp16.	Not reported.	Bouvet et al. (2012)
	Implicated in the regulation of polyprotein processing. Plays a pivotal role in viral transcription by stimulating both nsp14 3′-5′ exoribonuclease, and nsp16 2′-*O*-methyltransferase activities		
Nsp12	Responsible for replication and transcription of the viral RNA genome.	Not reported.	Ahn et al. (2012)
Nsp13	Magnesium dependent helicase activity.	Not reported.	Tanner et al. (2003)
	Displaying RNA and DNA duplex-unwinding activities with 5′ to 3′ polarity.		
Nsp14	An exoribonuclease acting on both ssRNA and dsRNA in a 3′ to 5′ direction.	Interacts with DDX1 via N-terminus.	Denison et al. (2011)
	Acts as a proofreading exoribonuclease for RNA replication.	Modulation of the innate immune response.	
Nsp15	Mn^2+^-dependent, uridylate-specific enzyme, which leaves 2′-3′-cyclic phosphates 5′ to the cleaved bond.	Essential to evade dsRNA sensors.	Ricagno et al., 2006)
Nsp16	Mediates mRNA cap 2′-O-ribose methylation to the 5′-cap structure of viral mRNAs.	Essential to evade MDA5 recognition.	Bouvet et al. (2010)
		Negatively regulating innate immunity.	
		IFN antagonism.	

### Virus Assembly and Egress

The structural proteins (Table 2) and some membrane accessory proteins are translated inside ER-bound ribosomes, while N protein is translated via free ribosomes in the cytoplasm (Nal et al., 2005). One of the distinct characteristics of CoVs is virion aggregation inside the ER and budding toward the Golgi apparatus (Cawood et al., 2007). Moreover, E, S, and N proteins interpolation to virions is modulated by M protein heterotypic interaction in the budding locus (Opstelten et al., 1995). After assembly and budding, the full virus particles are transferred to the cytoplasmic membrane and finally are released through the exocytosis process (Figure 1).

**TABLE 2:** The biological functions of structural proteins of SARS and SARS-CoV 2.

Protein	Post-translational modification	Biological functions in virus particle	Effect on the host cellular response	Refs.
S	Disulfide bridge, Palmitoylation, N-glycosylation	Virulence factor.	Physically interaction with eIF3F.	Bosch et al. (2003) and Nal et al. (2005)
		Responsible for recognition of the cellular receptor.	Modulation the expression of the pro-inflammatory cytokines IL 6 and 8 at a later stage of infection.	
		Fusion of virus membrane with host endosome membrane.		
M	O-glycosylation, N-glycosylation	Plays a central role in virus morphogenesis. Maintain the structure and assembly via its interactions with other viral proteins such as 3a and 7a, forms a complex with HE and S proteins.	Pro-apoptotic mediated activation of caspases 8 and 9.	Nal et al. (2005) and Siu et al. (2009)
		Promotes membrane curvature, binds to the nucleocapsid and participates in RNA packaging into the virus	Suppress IFN I production mediated by RIG-I and inhibiting the translocation of IRF3 into the nucleus, IFN antagonism	
E	Palmitoylation, glycosylation	Plays a central role in virus morphogenesis and assembly.	Induction of the cell stress response and mitochondrial-mediated apoptosis. Disruption of the lung epithelium, Potential B cell antagonism.	An et al. (1999) and Liu et al. (2007)
		Responsible for the curvature of the viral envelope.		
		A virulence factor trafficking within the infected cells and budding of the virion.		
N	*O*-glycosylation, ADP-ribosylation, Sumoylation, Phosphorylation	An RNA chaperone, associates with the viral genome in a helical nucleocapsid.	Modulate transforming growth factor-beta signaling by binding host smad3.	Zhao et al. (2006) and Wu et al. (2009)
		Plays a fundamental role during virion assembly through its interactions with the viral genome and membrane protein M, E and nsp3.	Interfere with the function of IRF3.	
		Plays an important role in enhancing the efficiency of sgRNA transcription and viral replication.	Inhibition of IFN I response. Induction of apoptosis.	

### Coronaviruses Pathogenesis

Clinically, the pathogenesis of coronaviruses is divided into three stages. In the viremia phase, the virus enters the peripheral blood after the lungs are infected. Thus, the virus can reach its target tissues, such as the heart, kidneys, and gastrointestinal tract, through the bloodstream. The second phase is the pneumonia phase. Then the patient enters the recovery phase if the immune system can overcome the virus’s attack at this stage. However, in patients with immunodeficiency, high blood pressure, diabetes, and the elderly, the immune system cannot effectively manage the infection, and a critical stage of the disease occurs (Peiris et al., 2003; Guan et al., 2020).

## Treatment

Vaccination is the best way to control the COVID-19 pandemic, and many efforts are being made to produce it. Nevertheless, its production is time-consuming because it must first be proven to be immunogenic and effective. Therefore, prevention is currently the best treatment. There are two essential strategies for treatment this disease. The first step is a general treatment that reduces the symptoms and clinical complications of the patients, and the second is drug treatment.

### General Treatment

The available treatment options for these patients include measuring and monitoring of vital signs such as heart function, kidney, liver, respiratory rate, and oxygen therapy if necessary. Moreover, patients must be getting enough water, sufficient calories, balance for electrolytes, and use of fever medicines such as acetaminophen and ibuprofen (Wang J. et al., 2020; Zimmermann and Curtis, 2020). Although the use of corticosteroids such as methylprednisolone in the SARS-CoV epidemic has been shown to improve some clinical symptoms, it is not common in COVID-19. Nevertheless, it could be used only temporarily in patients with severe illness such as dyspnea and Acute Respiratory Distress Syndrome (ARDS) (Huang et al., 2020; Russell et al., 2020; Zhang et al., 2020b). Herbal medicines have the property to be used as a dietary supplement for relieving and reduce respiratory symptoms and other symptoms of COVID-19. They can improve the general condition of patients with mild disease severity. *Althaea officinalis* L. (Malvaceae), *Commiphora myrrha* (T.Nees) Engl. (Burseraceae), *Glycyrrhiza glabra* L. (Fabaceae), *Hedera helix* L. (Araliaceae), and *Sambucus nigra* L. (Viburnaceae) are some of the herbal medicines can be used as adjunctive therapy for mild COVID-19 (Silveira et al., 2020).

### Coronavirus Drug Treatment

SARS-CoV-2 is a new virus from the Coronaviruses family, with 79% genomic similarity to SARS-CoV and 51.8% to MERS-CoV. Therefore, since the risk of SARS-CoV-2 vertical infection is the same as these two types of viruses, their treatment can be similar. Consequently, drugs that were previously effective for the treatment of SARS-CoV and MERS-CoV may also have therapeutic potential for the treatment of COVID-19 (Ren et al., 2020). Although a definitive cure for this new virus has not yet been discovered, previous studies suggest that drugs such as western medicines and natural products may have potential efficiency against COVID-19 and could be used to reduce the severity of the disease.

#### Interferon

Interferon type I is a member of the innate immune system produced and secreted in response to viral infections, including alpha and beta-interferon. Interferons exert their antiviral activity in two ways 1) cytotoxic T lymphocytes and macrophages stimulate the immune system to kill the virus by increasing or stimulating natural killer cells, 2) or inhibit virus replication in the host cell (Samuel, 2001; Sadler and Williams, 2008; Fensterl and Sen, 2009). Previous studies have shown that interferon-α, particularly its recombinant form (INF- α2b), can alleviate symptoms and shorten the disease course of respiratory tract viral infections in the early phase (caused by influenza, SARS-CoV, and MERS-CoV) through inhibiting virus replication and decreasing the virus load. Therefore, these molecules may be useful in the treatment of COVID-19 (Zheng et al., 2004; Danesh et al., 2011; Falzarano et al., 2013; Khalid et al., 2015). However, according to the latest update (3.2020) of the COVID-19 Treatment Guidelines Panel, there is no comment about its therapeutic effect on COVID-19 (COVID-19 Treatment Guidelines Panel).

#### Lopinavir/Ritonavir

Lopinavir/ritonavir is a protease inhibitor that has previously been used against HIV due to its antiviral activity. Nevertheless, later in the SARS-CoV (an epidemic that occurred in 2003), it was discovered that lopinavir/ritonavir by 3CL^pro^-inhibitor action could be useful for coronavirus treatment (Chu et al., 2004; Su et al., 2019). Due to the structural similarity of 3CL^pro^ in SARS-CoV2 and SARS-CoV, previous studies have suggested that this drug may be useful for treating SARS-CoV2 (Uzunova et al., 2020). Nevertheless, later it was found that these drugs do not have the desired effect in treating this infectious disease, and the coronavirus disease 2019 treatment guidelines (last updated: November 3, 2020) announced that its use is not recommended, except in a clinical trial (COVID-19 Treatment Guidelines Panel, 2019).

#### Ribavirin

Ribavirin is a nucleoside analog that has antiviral activity. Co-administration of lopinavir/ritonavir has a significant therapeutic effect against SARS-CoV and may reduce ARDS risk (Chu et al., 2004). However, according to the latest update (November 3, 2020) of the COVID-19 Treatment Guidelines Panel, there is no comment about its therapeutic effect on COVID-19 (COVID-19 Treatment Guidelines Panel).

#### Chloroquine

Chloroquine is an antiparasitic drug used against malaria, but recently it has been shown to have antiviral activity by blocking virus infection and could suppress SARS-CoV-2 infection *in vitro* (Wang M. et al., 2020; Colson et al., 2020). Although many physicians and specialists initially considered this drug to treat COVID-19, its use is no longer recommended today (COVID-19 Treatment Guidelines Panel).

#### Favipiravir

One of the promising drugs in the treatment of COVID-19 is favipiravir. This drug has an inhibitory effect on RNA polymerase, and its early clinical trial showed that not only is the antiviral activity of this drug more significant than lopinavir/ritonavir, but it has fewer side effects (Cai et al., 2020). However, according to the latest update (November 3, 2020) of the COVID-19 Treatment Guidelines Panel, there is no comment about its therapeutic effect on COVID-19 (COVID-19 Treatment Guidelines Panel).

#### Remdesivir

Remdesivir is one of the drugs that can be used against RNA viruses such as SARS-CoV/MERS-CoV. *In vivo* and *in vitro* studies have shown that this adenosine analog by inhibiting RdRP has a therapeutic effect against COVID-19 and could be a choice for treating this new pandemic infectious disease (Holshue et al., 2020; Wang M. et al., 2020). This drug is currently recommended to treat COVID-19 according to the latest update (November 3, 2020) of the COVID-19 Treatment Guidelines Panel (COVID-19 Treatment Guidelines Panel).

#### Arbidol

Arbidol is an antiviral compound for prophylaxis and treatment of influenza. An *in vitro* study has proved that arbidol has a direct antiviral activity on SARS-CoV replication (Wang X. et al., 2020). A clinical trial of this drug on SARS-CoV-2 has also shown that it reduces high flow nasal catheter (HFNC) oxygen therapy, enhances the process of viral clearance and improves focal absorption on radiologic images (Xu et al., 2020a). Arbidol could inhibit host cell adhesion and SARS-CoV-2 spike glycoprotein trimerization. Furthermore, the simultaneous use of arbidol by Lopinavir/ritonavir has a more significant therapeutic effect than the Lopinavir/ritonavir group (Deng et al., 2020). However, according to the latest update (November 3, 2020) of the COVID-19 Treatment Guidelines Panel, there is no comment about its therapeutic effect on COVID-19 (COVID-19 Treatment Guidelines Panel).

#### Antithrombotic Therapy

Anticoagulants, like heparins, play a vital role in preventing arterial thromboembolism in patients with heart arrhythmias. One of the leading causes of death in patients with COVID-19 is myocardial and stroke infarction for unknown reasons. Therefore, if hospitalized patients with COVID-19 due to hemophilia or similar diseases do not have anticoagulant contraindications, administration of a prophylactic dose plays a vital role in reducing the complications COVID-19 in patients with symptoms of blood coagulation (such as low levels of antithrombin, increased fibrinogen and di-dimer) (Godino et al., 2020).

### Natural Products as Antiviral Agents

**FIGURE 2:**
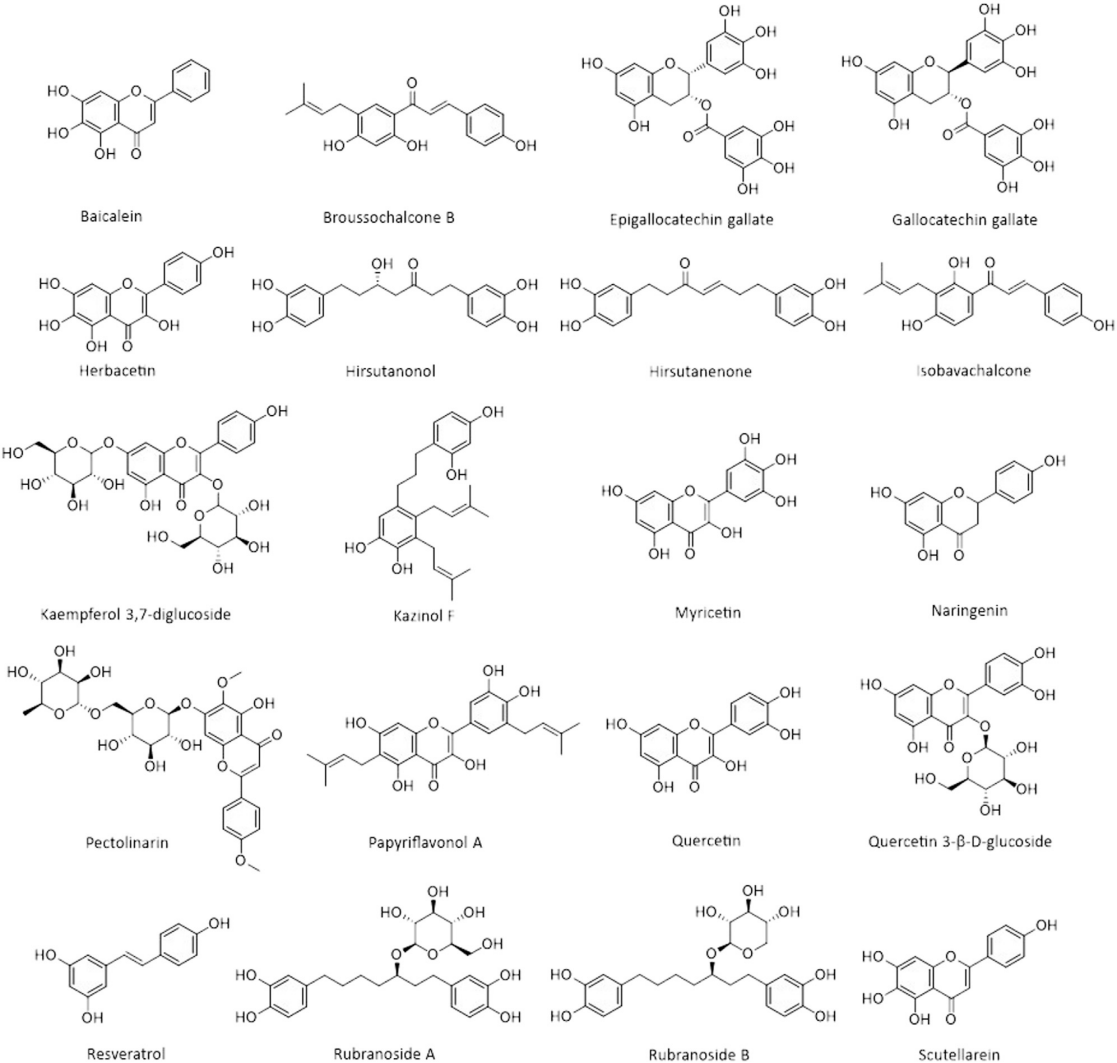
Chemical structures of some natural products introduced in this study.

**FIGURE 3:**
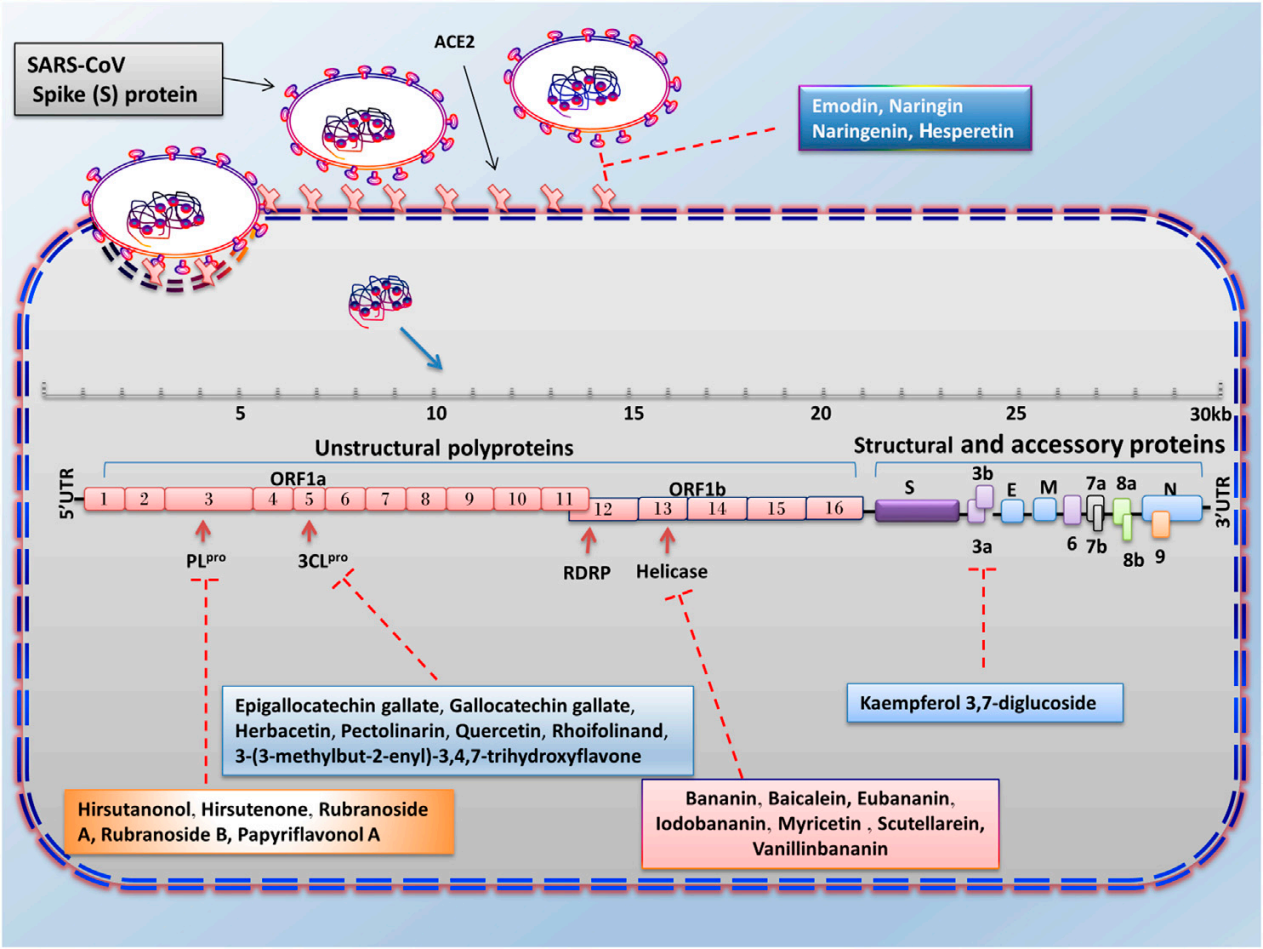
Advantages of employing natural compounds against infection and replication of SARS-CoV. Some of the natural compounds by reducing the expression of 3CL^pro^, PL^pro^, helicase, and 3a genes can play a therapeutic role in inhibiting the SARS-CoV infection.

**FIGURE 4:**
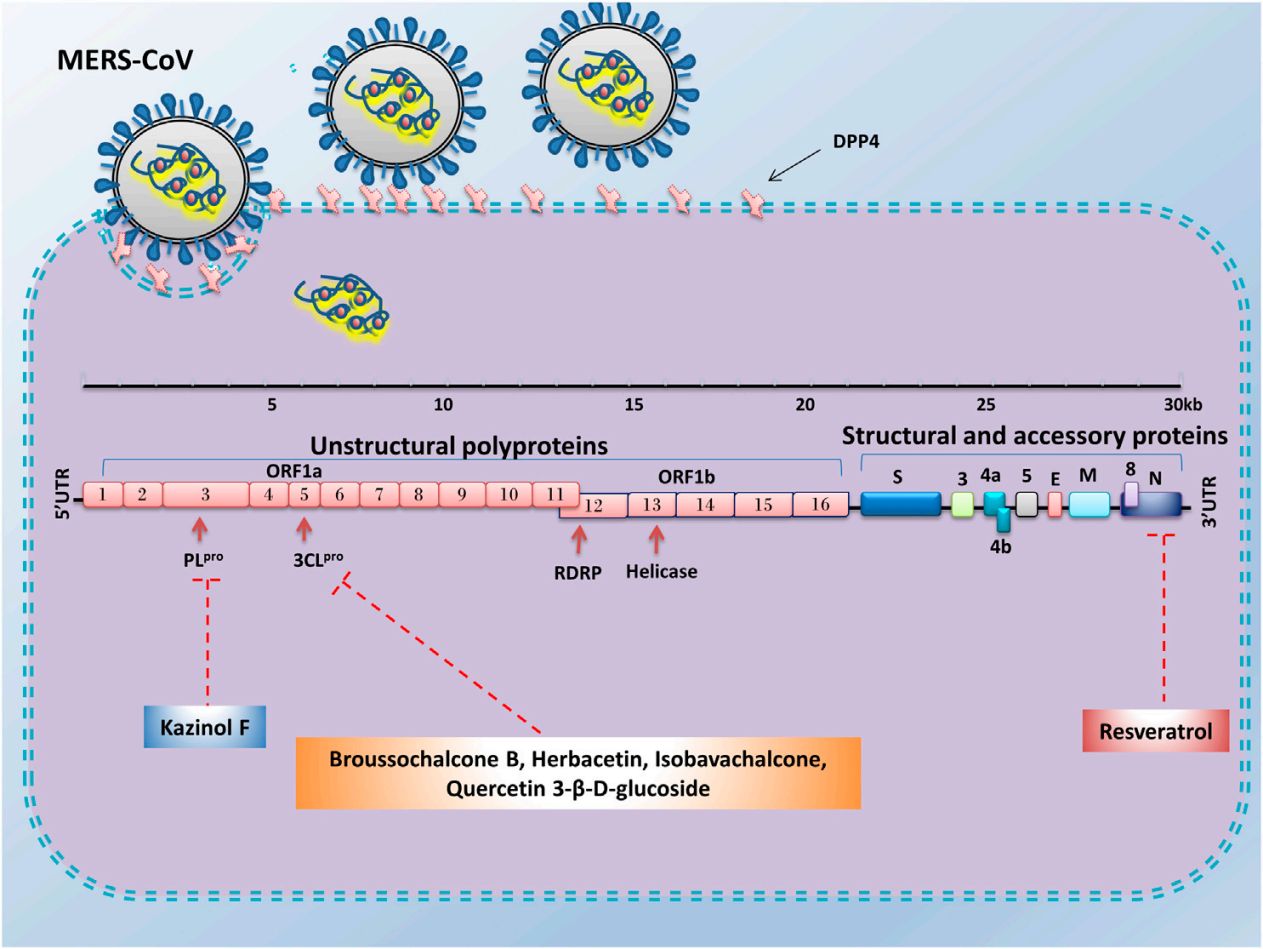
Advantages of employing natural compounds against infection and replication of MERS-CoV. Some of the natural compounds by reducing the expression of nucleocapsid, 3CL^pro^, and PL^pro^ genes could play a therapeutic role in inhibiting the MERS-CoV infection.

Natural products are biochemical mixtures produced by living organisms in nature The effectiveness of natural compounds in ancient medicine was introduced thousands of years ago (Ji et al., 2009). These compounds, with various pharmacological traits, have antioxidant, anticancer, anti-inflammatory, and antiviral activities. Today, many of the chemical drugs used in medicine are derived from natural compounds (Frabasile et al., 2017). The therapeutic strategy for natural products and chemical drugs is to target the protein molecules needed for each stage of the virus’s life cycle (Dias et al., 2012). This section discusses the natural compounds’ role in the inhibition of coronavirus infection (Table 3; Figure 2; Figure 3; and Figure 4). It is important to emphasize that within the compounds listed in Table 3, only epigallocatechin-3-gallate (NCT04446065) and resveratrol and zinc picolinate combination therapy (NCT04542993) has been investigated in clinical phase-2 trials against COVID-19 infection.

**TABLE 3:** Different types of phytochemicals by their sources and function in the coronavirus infection.

Compound name	Phytochemical class	Source	Function	Clinical trial stage	References
Baicalein	Flavonoid	*Scutellaria baicalensis* Georgi, *Scutellaria lateriflora* L. (Lamiaceae)	Inhibit 3CL^pro^ of SARS-CoV-1	—	Su et al. (2020)
			Decreased the levels of IL-1β and TNF-α in serum during the SARS-CoV-2 infection	—	Song et al. (2020)
Broussochalcone B	Polyphenol	*Broussonetia papyrifera* (L.) L’Hér. ex Vent. (Moraceae)	Inhibit PL^pro^ of SARS-CoV-1 and MERS-CoV	—	Park et al. (2017)
Curcumin	Polyphenol	*Curcuma longa* L. (Zingiberaceae)	Inhibit the ACE2 receptor and spike glycoprotein of SARS-CoV-2	—	Maurya et al. (2020).
Emodin	Anthraquinone	*Rheum palmatum* L. (Polygonaceae)	Inhibit the interaction of SARS-CoV-1 S protein and ACE2	—	Ho et al. (2007)
			Inhibit 3a protein channel of SARS-CoV-1	—	Schwarz et al. (2011)
Epigallocatechin-3-gallate	Polyphenol	*Camellia sinensis* (L.) Kuntze (Theaceae)	Inhibit 3CL^pro^ of SARS-CoV-1 and SARS-CoV-2	Phase 2 (SARS-CoV-2)	Jang et al., (2020) and Nguyen et al. (2012)
Gallocatechin gallate	Polyphenol	*Camellia sinensis* (L.) Kuntze (Theaceae)	Inhibit 3CL^pro^ of SARS-CoV-2	—	Jang et al. (2020)
			Inhibit PL^pro^ and CL^pro^ of SARS-CoV-1	—	Nguyen et al. (2012) and Park et al. (2016)
Herbacetin	Flavonoid	*Linum usitatissimum* L. (Linaceae)	Block the proteolytic activity of SARS-CoV-2 3CL^pro^	—	Jo et al. (2020a)
Hirsutanonol	Phenol	*Alnus glutinosa* (L.) Gaertn. (Betulaceae)	Inhibit PL^pro^ and 3CL^pro^ of SARS-CoV-1	—	Park et al., 2012a
Hirsutenone	Phenol	*Alnus japonica* (Thunb.) Steud. (Betulaceae)	Inhibits PL^pro^ activity of SARS-CoV-1	—	Park et al. (2012a) and Park et al. (2012b)
Iodobananin	Oligo-oxa-adamantane	Natural product derivative	Inhibit NTPase/Helicase of SARS-CoV-1	—	Tanner et al. (2005)
Isobavachalcone	Flavonoid	*Cullen corylifolium* (L.) Medik. (syn. *Psoralea corylifolia*) (Fabaceae)	Inhibit PL^pro^ of SAR-CoV-1	—	Kim et al. (2014)
Kaempferol 3,7- diglucoside	Glycosyloxyflavone	*Asplenium ruta-muraria* L., *Asplenium scolopendrium* L. (syn. *Asplenium altajense*) (Aspleniaceae)	Block virus ion channels in SARS-CoV-1	—	Schwarz et al. (2014)
			Target SARS-CoV2-S spike protein	—	Pan et al. (2020)
Kazinol F	Polyphenol	*Broussonetia papyrifera* (L.) L’Hér. ex Vent. (Moraceae)	Inhibit PL^pro^ of SARS-CoV-1 and MERS-CoV	—	Park et al. (2017)
Myricetin	Polyphenol	*Myristica fragrans* Houtt. (Myristicaceae)	Inhibit helicase and nsP13 of SARS-CoV-1	—	Yu et al. (2012)
Naringin	Flavonoid	*Citrus x aurantium* L., (Rutaceae)	Inhibit 3CL^pro^ of SARS-CoV-1	—	Nguyen et al. (2012)
Naringenin	Flavonoid	*Citrus* fruits (Rutaceae)	Two-Pore Channels (TPCs) inhibitor MERS-CoV	—	Pafumi et al. (2017)
			Block the enzymatic activity 3CL^pro^ in SARS-CoV-1	—	Jo et al. (2020b)
Pectolinarin	Flavonoid	*Cirsium chanroenicum* (Nakai) Nakai (Asteraceae)	Block the enzymatic activity of SARS-CoV-1 3CL^pro^	—	Jo et al. (2020b)
Papyriflavonol A	Flavonoid	*Broussonetia papyrifera* (L.) L’Hér. ex Vent. (Moraceae)	Inhibit PL^pro^ of SARS-CoV-1 and MERS-CoV	—	Park et al. (2017)
Quercetin	Glycosyloxyflavone	*Allium cepa* L. (Amaryllidaceae)	Target SARS-CoV2-S spike protein	—	Pan et al., 2020
Quercetin 3-β-D glucoside	Flavonoid	*Passiflora subpeltata* Ortega (Passifloraceae)	Inhibit 3CL^pro^ of MERS- CoV	—	Jo et al. (2019)
Resveratrol	Polyphenol	*Vitis vinifera* L. (Vitaceae)	Inhibit nucleocapsid protein translation in and reduced the MERS-CoV-mediated apoptosis	Phase 2 (SARS-CoV-2)^a^	Lin et al. (2017)
Rhoifolin	Flavonoid	*Toxicodendron succedaneum* (L.) Kuntze (syn. *Rhus succedanea*) (Anacardiaceae)	Inhibit 3CL^pro^ SARS-CoV-1	—	Nguyen et al. (2012)
Rubranoside A	Diarylheptanoids	*Alnus hirsuta* (Spach) Rupr. (syn. *Alnus sibirica*) (Betulaceae)	Inhibit 3CL^pro^ and PL^pro^ SARS-CoV-1	—	Park et al. (2016)
Rubranoside B	Diarylheptanoids	*Alnus japonica* (Thunb.) Steud. (Betulaceae)	Inhibit 3CL^pro^ and PL^pro^ SARS-CoV-1	—	Park et al. (2016)
Scutellarein	Flavonoid	*Scutellaria lateriflora* L. (Lamiaceae)	Inhibit helicase, nsP13 SARS-CoV-1	—	Yu et al. (2012)
Vanillinbananin	Oligo-oxa-adamantane	Natural product derivative	Inhibit NTPase/Helicase of SARS-CoV-1	—	Tanner et al. (2005)

^a^ Resveratrol and Zinc Picolinate combination therapy.

### Targeting S Protein, ACE2, and TMPRSS2 to Inhibit Cell Attachment and Entry

When a coronavirus enters the host cell, it must first, through its S protein, bind to the specific receptors at the surface of host cells. The particular receptor for SARS-CoV and SARS-CoV-2 is the ACE2 protein. To reduce the viral entry strength, the expression of the virus S protein can be inhibited or the expression of ACE2 in host cells can be reduced by natural products. Studies have shown that the amino acid sequence of SARS-CoV Spike protein is approximately 76.47%, similar to SARS-CoV-2 (Xu et al., 2020b). Since virus S protein is highly glycosylated, the use of plant-derived lectins, which tend to bind to glycosylated proteins, can prevent the virus from binding to receptors on the cell surface. Previous studies have shown that herbal compounds could be used as inhibitors for the human immune deficiency virus and SARS-CoV feline (Pyrc et al., 2007). Emodin belongs to the anthraquinones compounds (a group of natural compounds), which have anti-inflammatory, anticancer, and antioxidant activities (Izhaki, 2002). In a dose-dependent manner, emodin could significantly inhibit the ACE2 and S protein interaction in the biotinylated Enzyme-Linked Immunosorbent Assay (ELISA) method and Vero E6 cell line. Therefore, we can consider this compound as an antiviral therapeutic agent in the treatment of SARS-CoV (Ho et al., 2007). Some drugs and substances could reduce the expression of the ACE2 receptor. As seen, interleukin-4 and interferon-γ decreased this receptor in Vero E6 cells (De Lang et al., 2006). Molecular docking and structure-based drug design study reveal that berberine, thebaine, mangiferin, piperine, nimbin, and curcumin have an inhibitory effect against ACE2 receptor and spike glycoprotein of SARS-CoV-2. Among these, curcumin and nimbin have shown the highest interaction with ACE2 receptors and spike glycoprotein than other natural compounds and chemical drugs such as hydroxychloroquine, nafamostat, and captopril (Maurya et al., 2020).

The citrus peel is rich in flavonoid and alkaloid compounds (such as naringin, naringenin, hesperetin, and hesperidin) that play an essential role in maintaining gastrointestinal health and improving the immune system. Naringin is an anti-inflammatory substance that decreases the inflammatory effect in LPS-induced RAW 264.7 macrophages and LPS-treated rats. Therefore, it can inhibit the increase of pro-inflammatory cytokines such as IL-6, IL-1β, iNOS, and COX-2 induced by LPS treatment. Molecular docking investigation showed that naringin, naringenin, and hesperetin could bind to ACE2, such as chloroquine. Furthermore, these compounds can bind to ACE2 at lower binding energy levels than chloroquine (docking energy of 5.7 kcal/mol). However, more *in vitro* and *in vivo* experimentation is needed to determine whether these compounds are more effective than chloroquine (da Silva Antonio et al., 2020). However, it should be noted that inhibition of ACE2 due to its important physiological activities (such as homeostasis of blood pressure, protection against pulmonary cell destruction, electrolyte retention, and water) may have detrimental effects on patients’ life. In addition to ACE2, Transmembrane Protein Serine 2 (TMPRSS2) is another essential protein for SARS-CoV-1 and SARS-CoV-2 infection. TMPRSS2 induces viral fusion with the host cell membrane through irreversible structural changes in viral S protein (Hoffmann et al., 2020). As a result, the inhibition of TMPRSS2 in the animal model’s airways decreases the severity of lung damage after infection by SARS-CoV and MERS-CoV (Chikhale et al., 2020). Therefore, one of the strategies that could block the entry and restrict the pathogenesis of SARS-CoV-2 is the inhibition of TMPRSS2. The results of two independent studies using computational biology and molecular docking showed that some natural compounds such as neohesperidin, myricitrin, quercitrin, naringin, icariin, citicoline, bianthraquinone, isogemichalcone B, and (-)-epigallocatechin-(3-*O*-methyl) gallate could be used as a TMPRSS inhibitor (Chikhale et al., 2020; Rahman et al., 2020).

### Targeting Helicase and Inhibiting Virus Replication

SARS-CoV-2 Helicase protein is encoded by the nsp 13, a gene located downstream of the RdRP. The inhibition of this protein could reduce virus replication (Ivanov et al., 2004). Helicases could separate the double-stranded Nucleic Acid (NA) by using free energy that is obtained from the hydrolysis of Nucleoside Triphosphate (NTP). Therefore, this protein is a potential target for antiviral drug development. Previous studies revealed that various natural products could suppress helicase (unwinding or ATPase) activity. In 2005, Tanner et al. investigated the effect of adamantane-derived bananins on SARS-CoV helicase.

Their results showed that iodobananin, vanillinbananin, bananin, and eubananin had an inhibitory effect on the helicase protein’s ATPase activity with IC_50_ rates in the range 0.5–2.8 µM. Furthermore, using Fluorescence Resonance Energy Transfer (FRET) it was found that these four compounds could also inhibit unwinding helicase activity. The authors also observed that these compounds were ineffective against *E. coli* helicase but only affected SARS helicase, so they did not have a general helicase inhibitor activity. Finally, the results of cytopathic effects in fetal rhesus kidney-4 cells (FRhK-4) and RT-PCR demonstrated that these compounds could reduce virus replication without being toxic to the cell (Tanner et al., 2005). In 2012, the results of a study by Yu et al. showed that of the sixty-four natural compounds (flavonoids), only myricetin and scutellarein could reduce SARS-CoV helicase activity. The events showed that these two compounds could reduce Helicase ATPase activity by up to 90% at a dose of 10 µM without any toxicity effects on the healthy breast cell (MCF10A). Moreover, further analysis has shown that they do not change the helicase unwinding activity (Yu et al., 2012). Baicalein is another natural product that has an inhibitory effect on SARS-CoV (nsp13) helical activity. Studies have shown that this compound has no inhibitory effect on the dsDNA-unwinding activity of nsP13; however, it can decrease helicase ATPase activity up to 60% with an IC_50_ value 0.47 μM (Keum et al., 2013).

### Targeting 3CL^PRO^, PL^PRO^ Protease and Inhibiting Virus Protein Processing

Another important antiviral strategy is the use of specific inhibitors against viral proteases such as 3CL^pro^ and PL^pro^, which play an essential role in the processing and maturation of proteins and virus replication. The SARS-CoV-2 genome (positive single-stranded RNA), after translation, is capable of producing two polypeptides, pp1a and pp1ab. Finally, these two polypeptides, cleaved by 3CL^pro^ or PL^pro^ activity, produce sixteen non-structural proteins (Thiel et al., 2003; Muramatsu et al., 2016). Flavonoids are a group of phenolic compounds found in plants with anti-inflammatory, antiviral, antioxidant, and anticancer activities. There have been many reports of reduced coronavirus infection with these compounds (Tapas et al., 2008). *In vitro* studies in *Pichia pastoris* GS115 showed that epigallocatechin-3-gallate, quercetin, and gallocatechin gallate could reduce the expression of SARS-CoV 3CL^pro^. Molecular docking experiments and kinetic enzyme studies also revealed that these three compounds with IC_50_ rates in the range 47–73 µM could reduce protease activity up to 80%. In the meantime, gallocatechin gallate’s effect was more significant than the other compounds (Nguyen et al., 2012). Rhoifolinand, herbacetin, and pectolinarin are other flavonoid compounds that have an adverse influence on 3CL^pro^. Studies with FRET protease assays and absorption spectroscopic studies have shown that these compounds can bind to 3CL^pro^ and significantly inhibit its protease activity (drug concentration less than 40 µM in IC_50_) (Jo et al., 2020b). In another study, Jo et al. examined the inhibitory effects of several flavonoids compounds against MERS-CoV 3CL^pro^. Their results reveal the isobavachalcone, quercetin 3-β-D-glucoside, and herbacetin had noticeable inhibitory actions with IC_50_ values of 35.85, 37.03, 40.59 µM, respectively (Jo et al., 2019). The 3CL^pro^ of SARS-CoV-2 is highly conserved among all CoVs and has approximately 96% similarity with SARS-CoV-1. Due to its essential role in viral replication, it is a potential therapeutic target for COVID-19 (Xu et al., 2020c). Since natural products derived from microbial sources have a unique chemical diversity compared to plant products, more than 50% of FDA-approved natural compound-based medicines are derived from microbial compounds. The virtual screening and molecular binding by Sayed et al. showed that citriquinochroman, holyrine B, proximicin C, and several other microbial compounds could inhibit 3CL^pro^ of SARS-CoV-2 (Sayed et al., 2020). In addition to microbial compounds, molecular docking and MD simulation studies have shown that some marine natural products including hydroxypentafuhalol, pentaphlorethol B, and luteolin-7-rutinoside (ΔG about −14.6 to −10.7 kcal/mol) can also inhibit 3CL^pro^ of SARS-CoV-2 (Gentile et al., 2020).

The Papain-like protease (PL^pro^), is another protease that controls the proliferation of SARS-CoV-2 and is known as a potential target for treating this virus. Previous studies have shown that *Alnus japonica* (Thunb.) Steud. (Betulaceae) has anticancer, anti-inflammatory, and anti-influenza properties. The study of Kim et al. showed that the natural phenolic compounds (diarylheptanoids) prepared from this plant could change the proteolytic activity of PL^pro^. The fluorometric assay results showed that among the nine extracted substances, hirsutenone, hirsutanonol, rubranoside B, and rubranoside A had a dose-dependent inhibitory effect against PL^pro^. Among these compounds, hirsutenone had a remarkable inhibitory effect on SARS-CoV PL^pro^ (IC_50_ = 4.1 µM) and 3CL^pro^ (IC_50_ = 36.2 µM) enzyme activity. Further study showed that this substance, containing an α, β-unsaturated carbonyl group with a catechol moiety in the backbone, and the presence of this structure played an essential role in its inhibitory effects (Park et al., 2012a). In addition, polyphenols derived from the root of *Broussonetia papyrifera* (L.) L’Hér. ex Vent. (Moraceae) have been shown to have good inhibitory potential against SARS-CoV and MERS-CoV proteases. Park et al. investigated the inhibitory effect of ten natural compounds derived from this plant on SARS-CoV and MERS-CoV proteases. The effect of these compounds on SARS-CoV proteases showed that the papyriflavonol A (Broussonol E) was the most effective inhibitor of PL^pro^ (IC_50_ value 3.7 µM) and 3-(3-methylbut-2-enyl)-3,4,7-trihydroxyflavone was the most useful inhibitor of 3CL^pro^ (IC_50_ value of 30.2 µM). Additionally, broussochalcone B (Bavachalcone), with a concentration of 27.9 µM (IC_50_) and kazinol F with a concentration of 39.5 µM (IC_50_), were able to reduce the activity of MERS-CoV 3CL^pro^ and MERS-CoV PL^pro^, respectively (Park et al., 2017).

### Targeting Nucleocapsid (N) Protein to Inhibit Virus Infection and Replication

Resveratrol is a natural compound with anti-inflammatory, antioxidant, and anticancer properties (Yeung et al., 2019; Filardo et al., 2020). The previous study demonstrated that this compound has antiviral activity and can inhibit viral infections caused by Herpes Simplex Virus (HSV), Respiratory Syncytial Virus (RSV), and Epstein-Barr Virus (EBV) (Faith et al., 2006; Zang et al., 2011; De Leo et al., 2012). Also, resveratrol by repressing the expression of MERS-CoV N protein in the Vero E6 cell line, could reduce RNA expression, viral yield, and replication of MERS-CoV. Additionally, it significantly decreases the virus’s infection and enhances infected cells’ survival by repressing Caspase 3 cleavage (Lin et al., 2017). Other natural compounds that can reduce coronavirus infection risk include the alkaloids fangchinoline, cepharanthine, and tetrandrine. The results of a study by Kim et al. showed that these compounds could inhibit the expression of pro-inflammatory cytokines (IFN-α1, IL-6, IFN-β1, IL-8, and IL-1) caused by human coronavirus OC43 infection in the MRC-5 cell line. These three natural compounds can also decrease the OC43 replication by inhibiting N protein expression and reducing the cytotoxic effect of this virus in the MRC-5 cell line, and increasing the proliferation and survival of MRC-5 human lung cells (Kim et al., 2019).

### Targeting 3A Protein and Inhibiting Virus Release

The production and release of the virus require some ion channels in the host cell membrane. Therefore, inhibition of these ion channels played a significant role in inhibiting viral infections, so one antiviral strategy is to use compounds that can restrain these channels (Liang and Li, 2010). One of these ion channels is the cation-selective channel (3a protein) generated by the ORF3a of the SARS-CoV genome. Kaempferol glycoside is a natural flavonol found in a variety of plants. Previous studies have shown that this compound has antiviral properties (IC_50_ value of 2.3 μM) and can inhibit the expression of 3a protein SARS CoV (an ion channel) in *Xenopus* oocyte as a model system (Schwarz et al., 2014).

## RNA Interference

**FIGURE 5:**
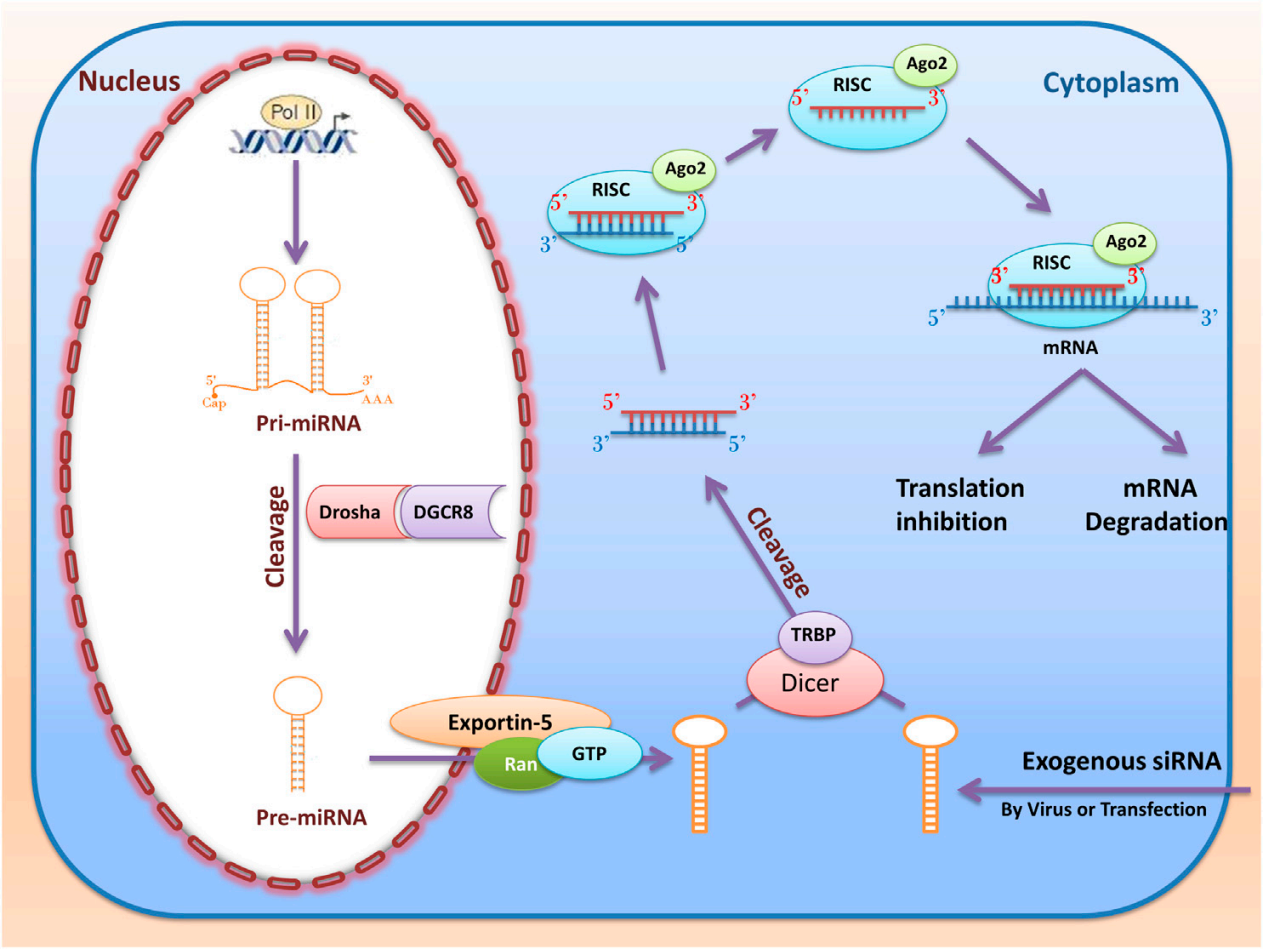
RNA interference (miRNAs and siRNAs) biogenesis and function. The miRNAs first transcribed from the nucleus genome as pri-miRNA. Then pri-miRNA cleavage with Drosha and DGCR and converted to pre-miRNA. Then, RanGTP and exportin 5 cause the pre miRNA to be transported from the nucleus to the cytoplasm and cleavage by Dicer and TRBP. Ultimately, after entering the RISC complex, mature miRNA can be attached to the target mRNA and perform their function by destroying mRNA or inhibiting the translation. When inserting into the cell, synthetic siRNAs are cleaved by Dicer in the cytoplasm. This dsRNA enters RISC, and if cold finds the target mRNA, the mRNA is cleaved by the RISC and Ago2.

RNA interference (RNAi) are RNA molecules found in many eukaryotes that inhibit gene expression by targeted 3UTR of mRNA molecules. Today, siRNA and miRNA are the most common type of RNAi used for gene silencing. RNAi origin can be endogenous (originating in the cell) and exogenous (coming from a virus or laboratory tools). Exogenous RNAi can be transmitted to cells using electroporation, viral vectors, liposomes, and calcium phosphate (Sohrab et al., 2018). When inserting into the cell, synthetic siRNAs are cleaved by Dicer in the cytoplasm. After placement in the RNA-Induced Silencing Complex (RISC) and Ago2, RNAi molecules (Figure 5) become single-stranded RNA and can destroy the target mRNA or inhibit its translation (Ding et al., 2018).

### The Therapeutic Effect of siRNA on Coronaviruses

**FIGURE 6:**
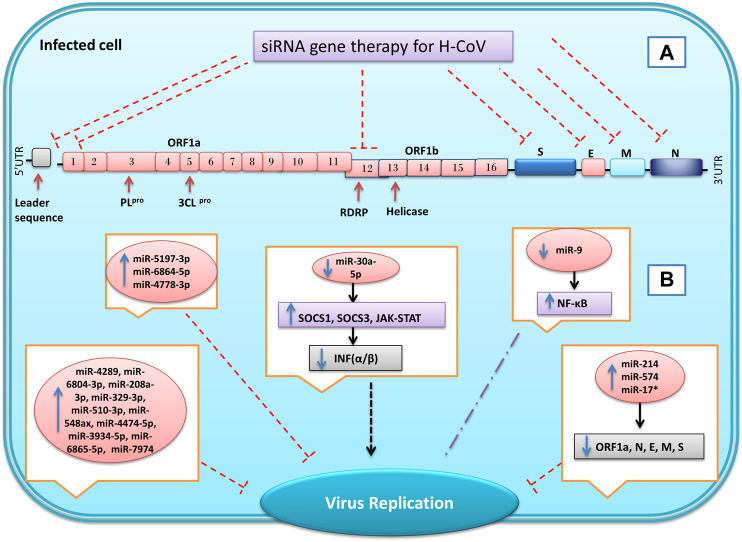
Inhibition of genes expression of SARS coronavirus using miRNAs and synthetic small interfering RNAs (siRNA). By designing specific siRNAs, it is possible to inhibit the expression of the virus’s structural and non-structural genes for reduce the replication and infusion of the virus **(A)**. In coronavirus infection, the microRNAs expression of host cells changes in response to infection. Some of these changes are the cell’s response to the infection, and others are caused by the virus, which can eventually lead to a reduction or increase in virus replication and infection **(B)** Inhibition 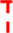, Induction 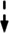, not define 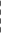, Up-regulation 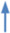, Down-regulation 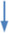.

Synthetic siRNAs have about 21–23 bp length and perform their role by inhibiting gene expression at the post-transcription level. Unlike miRNAs, each siRNA is designed against a specific gene, so it can only impede that gene expression (Taxman et al., 2006). The siRNAs are first inserted into the cell as long double-stranded RNAs and then are cleaved by RNase III (Dicer) in the cytoplasm to become small dsRNAs of approximately twenty-one base pairs. This dsRNA later enters the RISC and converts to single-stranded RNA (ssRNA). If RISC and siRNAs complex could find the specific target site on mRNA, it could cleave the mRNA, and cellular exonucleases could invade to destroy the target mRNA (Wu and Chan, 2006). Studies have shown that this type of RNAi has the potential to act as antiviral agent to reduce replication and infection of many viruses such as HIV, Flock House Virus (FHV), Hepatitis C Virus (HCV), and Hepatitis B Virus (HBV) (Hamasaki et al., 2003; Liu et al., 2017; Shahid et al., 2017; Taning et al., 2018). Previous studies have shown that siRNA could target different parts of the virus genome to reduce the replication and infection of SARS-CoV (Figure 6A). For example, targeting the nsp1 gene (from nucleotides 250–786) by siRNA is one of the best ways to control SARS-CoV. Because targeting this area of the genome inhibits virus propagation, pathogenesis, and replication in Vero E6 cells (Ni et al., 2005). Another strategy that can help to suppress infection and replication of SARS-CoV is inhibiting the virus S protein or its specific receptor ACE2.

The SARS-CoV Spike protein is an essential viral surface glycoprotein for identifying target cells and interacting with ACE2 host cell receptors (Gallagher and Buchmeier, 2001). A study by Zhang et al. reveals that inhibiting expression of S protein using specific siRNAs could reduce the viral titers, infection, and replication of SARS-CoV in Vero E6 and 293T cells (Zhang et al., 2004). RNAi technology is also a useful instrument to suppress ACE2 expression at the host cells’ surface to counteract SARS-CoV infection. In addition to the lungs, ACE2 is expressed on the surface of the bronchial, renal, duodenum, colon, gastrointestinal, and cardiovascular cells (Donoghue et al., 2000). Inhibition of the ACE2 protein in Vero E6 cell lines using siRNA could reduce replication, copies number, and infection of severe acute respiratory syndrome-associated coronavirus (Lu et al., 2008). Li et al. investigated the effect of siSC5 (nsp12 region) and siSC2 (spike protein) against SARS-CoV on *in vitro* and *in vivo* models. Their results showed that these two siRNAs by inhibited virus replication in FRHK-4 cells could reduce the virus infection’s effects and symptoms without having any toxicity effect on *Rhesus macaque* (dosages of 10–40 mg/kg) (Li et al., 2005a).

The spike protein structure determines the type of host cells which could be infected by a virus. However, variations of S protein are seen among the various strains of coronavirus. Therefore, one of the essential strategies for controlling viral infections targets the conserved genome areas between various coronaviruses (Kuo et al., 2000). Hence, Wang et al. tried to use two specific siRNAs against the conserved sequence of the SARS-CoV. The study results showed that by targeting two regions (14,450–14,468 and 15,877–15,895), encoding RNA polymerase could inhibit expression of RNA polymerase, N protein, 3CL^pro^ and also reduce replication and cytopathic effect of the virus (Wang et al., 2004). Among the two COVID-19 proteases, the nsp3 sequence encoding papain-like protease is less conserved, while the nsp5 sequence encoding 3CL^pro^ is highly conserved and can be selected as a potential target of siRNA for COVID-19 treatment (Liu et al., 2020).

The RdRP gene encodes a key enzyme for the replication of the virus. In SARS-CoV-2, this gene is located in ORF1b by 645 bp long and has been reported to be highly conserved, so it has the potential to target several siRNAs. Also, investigations of the RdRP sequence have shown that this gene has no genetic similarity to human genes and other coronaviruses (Wu et al., 2020). Lu et al.’s investigation shows using specific siRNA can reduce SARS-CoV RdRP expression by more than 90% in HeLa and 239T cell lines, leading to inhibition of plaque formation in Vero E6 cells. Therefore, its suppression could be considered a suitable therapeutic target in COVID-19 patients (Lu et al., 2004). The siRNAs could target other essential coronavirus genes. For example, by designing three different siRNAs, Shi et al. were able to reduce the expression of N, E, and M genes of SARS-CoV in Vero E6 cells (Yi et al., 2005). Furthermore, the replication of SARS-CoV could be inhibited by targeting the leader sequence using specific siRNA in Vero E6 cells (Li et al., 2005b).

Although previous studies have shown that RNAi has antiviral potential, it appears that viruses can use these molecules to their advantage. MERS-CoV could infect both human and bat cell lines. Nevertheless, its pathogenic power and replication are varied between the bat (*Eptesicus fuscus*) cells and human (A549, MRC5, and Huh7) cell lines. In human cells, MERS-CoV shut-down interferon antiviral responses in the innate immunity system, unlike in bat cells by inhibition of IRF3 (a critical activator for INF β expression) (Banerjee et al., 2019).

Complemented palindromic small RNAs (cpsRNAs) are a group of small RNAs produced by mammalian and invertebrate viruses. There are sequences in the SARS-CoV (ORF3b) genome that have the origin of a cpsRNA called SARS-CoV-cpsR-19. The apoptosis assay events indicate that SARS-CoV-cpsR-19 could induce apoptosis in HeLa cells by increasing the caspase 3 and BAX/BCL2 ratio and may play an essential role in SARS-CoV pathogenesis (Liu et al., 2018). It has been shown that some viruses (such as the Ebola virus and influenza A) could preserve themselves from RNAi-based immune systems and facilitate their replication by using Viral Suppressors of RNA silencing (VSR). Studies of Cui et al. have shown a short hairpin RNA (a novel VSR) in the SARS-CoV nucleocapsid protein sequence that defeats RNAi-triggered suppression (Cui et al., 2015). Moreover, N protein overexpression in Neuro-2a cells efficiently inhibits Dicer-mediated dsRNA cleavage and could increase replication and titration of MHV-A59 (a close relative to SARS-CoV in Coronaviridae family) viruses. The SARS-CoV-2 (N) nucleocapsid protein can also act as an escape agent from the immune system and contribute to its pathogenicity. N proteins have high homology (94%) of the amino acid sequences among coronaviruses. A recent study has shown that the N protein of SARS-CoV-2 has VSR activity that can antagonize RNAi in both effectors (recognition and cleavage of viral dsRNA by Dicer) and initiation (siRNA biogenesis) steps (Mu et al., 2020).

### MicroRNAs

The miRNAs are a group of small non-coding RNAs with twenty-two nucleotides of length. These molecules are first transcribed from the nucleus genome (exons, introns, and intergenic regions) as pri-miRNA with a length of two hundred nucleotides to several thousand nucleotides (Booton and Lindsay, 2014). It is noteworthy that each pri-miRNA can be a precursor to several mature miRNAs. Then, first processing of pri-miRNA starts with RNase III (Drosha) and its cofactor (DGCR) to form pre-miRNA, a hairpin with a length of about sixty nucleotides. In the next step, RanGTP and exportin 5 cause the pre-miRNA to be transported from the nucleus to the cytoplasm. Dicer and TRBP performed the second processing in the cytoplasm to create a double-stranded RNA molecule with a length of about twenty-two nucleotides (Bartel, 2004). Ultimately, after entering the RISC complex, one of the strings (passenger strand) is destroyed, and mature miRNA (guide strand) can be attached to the target mRNA. MiRNAs perform their function by destroying mRNA or inhibiting the translation (Vazquez, 2006). Previous studies have shown that a miRNA alone can regulate the expression of several different genes by its function. Meanwhile, various miRNAs can simultaneously control the expression of one mRNA. However, about 60% of human genes can be regulated by these molecules (Kalhori et al., 2020). For the first time, Lecellier et al. (2005) reported that cellular miR-32 could reduce virus replication of the primate foamy virus (PFV-1) by targeting the viral RNA genome (Lecellier et al., 2005).

#### MicroRNA Regulates the Innate Immune System, Virus Replication, and Pathogenesis of Coronavirus

The innate immune system is the body’s first defense against viruses and bacteria. This system’s principal cells include macrophages, dendritic cells, natural killer cells, monocytes, and granulocytes (Leon-Icaza et al., 2019). Viruses, to increase replication, suppress the host’s innate immune system via reducing INF α/β production. For example, Japanese Encephalitis Virus (JEV), Dengue Virus (DENV), and Enterovirus 71 (EV71) are able to inhibit the overexpression of INF α/β in response to viral infection by enhancing the expression of miR-146a in infected cells (Wu et al., 2013; Ho et al., 2014; Sharma et al., 2015). Viruses can also decrease the innate immune system by inhibiting the expression or function of some miRNAs. For instance, in oligodendroglioma cells, the Borna Disease Virus (BDV) can inhibit the expression of miR-155 by its specific phosphoprotein, thus inhibiting INF α/β overexpression in response to viral infection and reducing the innate immune system (Zhai et al., 2013). Therefore, one of the main antiviral components of the intrinsic immune system is type 1 interferons (INF α/β).

Coronaviruses can prevent the induction of the immune system in response to viral infection with different strategies. *In vivo* and *in vitro* studies of Ma et al. on the Transmissible Gastroenteritis Virus (TGEV), a member of the *alpha-coronavirus* family, showed that this virus could downregulate miR-30a-5p expression. Furthermore, they found that virus replication was facilitated by reducing IFN-I signaling cascades via removing the inhibitory effect of miR-30a-5p on INF negative regulators (such as SOCS1, SOCS3, and JAK-STAT) (Ma et al., 2018). Therefore, the overexpression of miRNAs in infected cells could increase the innate immune system and may be considered as a therapeutic approach for treatment.

Although it has previously been reported that miRNAs play a significant role in regulating the eukaryotic gene, subsequent studies showed that these nano molecules can also alter the virus’s replication to increase or decrease its infection (Figure 6B). For example, miR-122 plays an essential role in the pathogenesis and replication of the Hepatitis C virus. The miR-122, to increase the virus’s stability and replication bind to the 5′ non-translated regions (NTRs) of the virus and repress RNA degradation via exonucleases. Therefore, the knockout of this miRNA in Huh-7 cells could reduce HCV replication (Jopling et al., 2005). In contrast, miR-32 could negatively alter the replication of the PFV-1 by targeting viral genes in the human HEK-293T cell line (Lecellier et al., 2005).

Some viruses have sequences (hairpin) in their genomes similar to miRNAs and can regulate the gene expression of the host cell or virus (Grundhoff and Sullivan, 2011; Kincaid and Sullivan, 2012). A computational approach study by Hassan et al. showed there are several hairpins in the genome of the MERS that could act as a precursor for thirteen miRNAs, which were significantly similar to human miRNAs. Their study showed ten miRNAs (miR-4289, miR-6804-3p, miR-208a-3p, miR-329-3p, miR-510-3p, miR-548ax, miR-4474-5p, miR-3934-5p, miR-6865-5p, and miR-7974) of these, miRNAs do not have any known specific biological function in humans or animals at all. Nevertheless, miR-18a, miR-628, and miR-342-3p had a biological role in humans related to Basal Cell Carcinoma (BCC) of the skin, malignant glioblastoma, and late-stage prion disease, respectively (Hasan et al., 2014). Numerous studies and reports suggest that some miRNAs have antiviral activity and can be used against influenza, HIV, HBV, and poliovirus (PV) (Sanghvi and Steel, 2012; Zhang et al., 2013; Shim et al., 2016; Hamada-Tsutsumi et al., 2019). On the other hand, viruses could alter the gene and miRNA expression profile in the host cell. For example, miR-146a and miR-130b upregulated by the human T Cell Leukemia Virus (HTLV-1) in PBMC cells (Bouzar and Willems, 2008).

Nucleocapsid (N) protein is a structural protein that has the same function in all coronaviruses. The human coronavirus CoV-OC43 could inhibit miR-9 function by its N protein and increase NF-κB expression in 253T cells. However, it is unclear whether upregulation NF-κB is a suitable response for virus replication or a secondary inhibitor for virus replication (Lai et al., 2014). Infection of bronchoalveolar stem cells (BASICs) by SARS-CoV reveals that this virus could upregulate the expression of miR-574-5p, miR-214, and miRNAs-17* 2–4 fold. Moreover, overexpression of these miRNAs could repress SARS-CoV replication by targeting the four viral structure proteins (E, S, M, N), and orf1a (Mallick et al., 2009). Unfortunately, to date, not many *in vivo* and *in vitro* studies have been performed on RNAi’s role in inhibiting the COVID-19, and most studies have been performed base on bioinformatics and *in silico* studies. Qingfei Paidu decoction (QFPD) contains twenty-one traditional Chinese medicines that have been used to treat COVID-19 since February 7, 2020. Chen et al.’s molecular docking study revealed that QFPD can bind to structural and non-structural proteins of COVID-19. They also found that miR-183 and miR-130A/B/301 predict targets of QFPD, and QFPD by these microRNAs may exert anti-SARS-CoV-2 activity (Chen et al., 2020a). In a bioinformatics approach study performed by Khan et al., it was found that several miRNAs can have antiviral properties in infections caused by SARS-CoV-1 and SARS-CoV-2. For example, evidence has shown miR-323a-5p, miR-622, miR-198, and miR-654-5p for SARS-CoV-1 and miR-323a-5p, miR-20b-5p, miR-17-5p for SARS-CoV-2 have antiviral roles by targeting the ORF1ab and the S region (Khan et al., 2020).

Therefore, it is likely miRNA with low side effects can be used as a therapeutic agent for the COVID-19 treatment. Differences in miRNA expression profiles in individuals are probably one reason why COVID-19 causes death in some people and causes only brief symptoms in others. As a result, microRNAs have recently emerged as a critical factor in increasing or inhibiting the potential of viral infection. We hope that clinical and preclinical research can use them in gene therapy as antiviral agents soon.

## Conclusion

Today, the whole world is suffering from a pandemic disease called COVID-19, which has caused deaths in many developed and developing countries. Despite all the advances in human medicine, we have not yet been able to find a suitable treatment for this viral disease. The use of molecular or pharmacological methods to control infection or virus replication requires identifying essential genes involved in infection and replication of the virus. Two strategies are suggested to treat this disease. The first step is to reduce the virus’s infection by preventing the virus from attaching to its specific receptor. The next step is to reduce the virus’s replication by inhibiting the virus’s structural and non-structural genes. Medicinal plants and natural products are a good option for preventing and treating viral infections, especially COVID-19, due to their lower cost, lower side effects, and natural origin compared with chemical drugs. These compounds could increase efficiency and strengthen the host immune system against many infections and diseases due to their inherent properties. In the treatment of COVID-19, these compounds can reduce virus infection or replication by repressing the virus’s coupling to the host cell’s receptors or by inhibiting the expression of structural and non-structural genes. Moreover, RNAi (siRNA and miRNA) could inhibit viral infections, especially COVID-19, by inhibiting essential virus genes or inducing a host immune system. Therefore, the simultaneous use of natural compounds and RNAi can play a critical role in the treatment of SARS-CoV-2 and restraining this pandemic pneumonia.
